# Epigenetic adaptations and vulnerabilities in ovarian cancer drug-tolerant persister cells

**DOI:** 10.1093/narcan/zcag030

**Published:** 2026-10-22

**Authors:** Mihai G Dumbrava, Sierra J Hamernick, Shariska P Harrington, Alexandre Gaspar-Maia

**Affiliations:** Division of Investigative Pathobiology, Department of Lab Medicine and Pathology, Mayo Clinic, Rochester, MN 55905, United States; Mayo Medical Scientist Training Program, Mayo Clinic, Rochester, MN 55905, United States; Division of Investigative Pathobiology, Department of Lab Medicine and Pathology, Mayo Clinic, Rochester, MN 55905, United States; Mayo Clinic Graduate School of Biomedical Sciences, Mayo Clinic, Rochester, MN 55905, United States; Department of Obstetrics and Gynecology, Mayo Clinic, Rochester, MN 55905, United States; Division of Investigative Pathobiology, Department of Lab Medicine and Pathology, Mayo Clinic, Rochester, MN 55905, United States

## Abstract

High-grade serous ovarian carcinoma (HGSOC) recurs in most patients despite initial responses to chemotherapy and poly(ADP-ribose) polymerase inhibition. A leading explanation for the high rates of recurrence is the presence of minimal residual disease (MRD) comprised of drug-tolerant persister (DTP) cells that survive therapy through reversible epigenetic reprogramming. DTPs have various adaptations that enable them to tolerate therapy and eventually develop into resistant clones, including quiescence, metabolic and hypoxic stress responses, epithelial-mesenchymal plasticity, and immune evasion. Here, we discuss evidence from ovarian cancer of epigenetic mechanisms that coordinate DTP phenotypes, including DNA methylation, histone post-translational modifications, enhancer rewiring, and transcription factor gene-regulatory networks. Based on therapeutic vulnerabilities identified from increased resolution of DTPs, we highlight epigenetic therapies that are currently in clinical trials aimed at depleting MRD to delay or prevent relapse in HGSOC. Finally, we outline emerging technologies that have the potential to detect and monitor persister states in patients, including single-cell transcriptomic and epigenomic profiling, long-read sequencing, and spatial mapping of tumor biopsies.

## Graphical Abstract

**Graphical Abstract:**
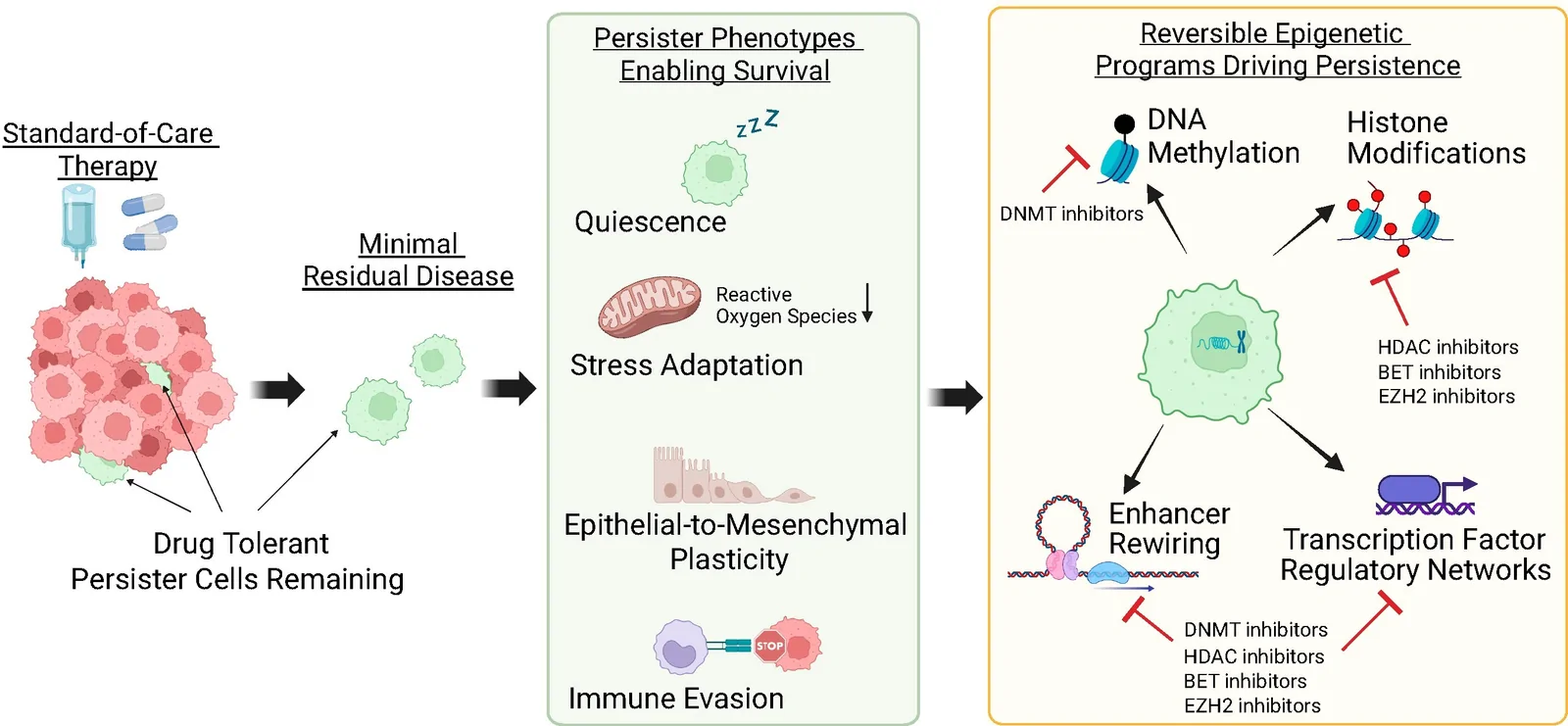

Impact statement The most common type of ovarian cancer (high-grade serous ovarian cancer) often recurs despite an initial response to treatment. This review highlights emerging evidence that recurrence is driven, not only by genetic alterations in tumor cells, but also by reversible epigenetic changes that enable populations of cancer cells to survive therapy. It describes how epigenetic adaptations affecting cell-cycle regulation, metabolism, and interactions with the tumor microenvironment contribute to drug tolerance, and it identifies potential therapeutic strategies targeting these survival mechanisms. Advances in single-cell and spatial technologies may accelerate the identification of biomarkers of persister cells and the development of more effective strategies to prevent ovarian cancer recurrence.

## Introduction

Advanced-stage high-grade serous ovarian carcinoma (HGSOC) is one of the most lethal gynecologic malignancies, with a five-year survival rate below 30% [1]. At the time of detection, most HGSOC patients present with advanced stage disease (III/IV) and will receive a frontline treatment regimen consisting of radical debulking surgery combined with adjuvant or neoadjuvant platinum and taxane chemotherapy [1, 2]. Following frontline treatment, maintenance strategies to delay the time to recurrence are selected based on tumor homologous recombination deficiency (HRD) status, response to platinum-based chemotherapy, and systemic toxicity. Of these, poly(ADP-ribose) polymerase (PARP) inhibitors, such as olaparib or niraparib, are approved as maintenance therapies for patients with ovarian cancer who respond to platinum-based chemotherapy, with the greatest clinical benefit observed in tumors harboring HRD, including *BRCA1/2* alterations [3]. Bevacizumab may also be incorporated into maintenance strategies, particularly in patients without confirmed BRCA1/2 mutations [4]. Recently, the development of antibody-drug conjugates (ADCs) has emerged as a promising therapeutic strategy, especially for platinum-resistant HGSOC, through targeted delivery of a highly potent cytotoxic payloads to tumor cells [5–7]. Although ADCs aim to minimize the systemic toxicity of conventional chemotherapy through targeted delivery of cytotoxic agents, their efficacy can be limited by heterogeneous target antigen expression within tumors and insufficient specificity for malignant cells [5–7].

Despite initially high response rates in most patients and recent therapeutic advancements, HGSOC recurrence is common. Approximately 75% of patients with advanced-stage disease will relapse within 18 months following the completion of first-line therapy even when a complete clinical response is achieved [8, 9]. Notably, the mechanisms underlying resistance are also shaped by the specific therapeutic context in which they arise. Resistance to the frontline regimen of platinum-based chemotherapy has been linked to epigenetic alterations in histone modifications and DNA methylation [10, 11]. Resistance to PARP inhibitors (PARPi) involves restoration of the HR pathway through *BRCA1/2* reversion mutations and the loss of *BRCA1* promoter methylation [12, 13]. Bevacizumab resistance is mediated in part by activation of alternative pro-angiogenic signaling pathways and remodeling of the tumor microenvironment, whereas ADC resistance can arise from downregulated target antigen expression [6, 7, 14]. Recurrent disease is stratified by the platinum-free interval, defined as the time from the last dose of platinum-based chemotherapy to the onset of recurrence. With each successive remission and subsequent relapse, the platinum-free intervals shorten, and most patients develop resistance [15]. Thus, to treat advanced ovarian cancer, there is a need for strategies that eradicate minimal residual disease (MRD), defined as clinically undetectable residual cancer cells that persist through treatment and seed recurrence [9]. Despite the different selective pressures imposed by each therapeutic regimen, there is a need to identify common targetable mechanisms underlying drug tolerance in MRD.

HGSOC represents a valuable model to study features of tumor chemotherapy responses since the standard of care is generally uniform across patients. The majority of HGSOC tumors are characterized by somatic mutations in the tumor suppressor gene *TP53* (>95% of cases), and ~50% exhibit HR DNA repair deficiency arising from either pathogenic variants (germline, somatic, or reversion mutations) in HR genes or from epigenetic silencing, most notably *BRCA1* promoter methylation [12, 16]. High levels of *BRCA1* promoter methylation are predictive of HRD status and inform assessment of platinum and PARPi sensitivity [12, 13]. HGSOC is further characterized by extensive copy number variation (CNV) and one of the highest whole-genome doubling (WGD) rates found in primary tumors [17]. Common amongst epithelial ovarian tumors, Cyclin E1 (*CCNE1*) copy number amplification has been associated with poor overall survival in HGSOC, especially when paired with an intact HR system [18–20]. Notably, despite the extensive chromosomal instability in these malignant cells, few genes aside from *TP53* and *BRCA1/2* are mutated in >1% of HGSOC tumors [21]. This underscores the critical role of non-genetic mechanisms as drivers of ovarian cancer progression and therapeutic resistance [10, 11, 17, 20, 22–25]. These epigenetic features modulate transcriptional programs and cellular functions without modifying the underlying DNA sequence.

Epigenetic plasticity, the dynamic remodeling of chromatin, has been proposed as a driver for the ability of a subset of cancer cells to adopt a drug-tolerant persister (DTP) state [24, 26]. This subpopulation of cells, which is derived from multiple lineages, survives lethal therapy through reversible transcriptional and epigenetic changes rather than stable mutations [24, 26]. Altered chromatin accessibility promotes adaptative mechanisms such as quiescence and metabolic rewiring in persisters that enable them to tolerate the course of therapy. Once treatment regimens cease, these cells, which comprise MRD, can reversibly regain their proliferative capacity, resulting in recurrent disease. A subpopulation of these DTPs will develop into resistant clones, leading to chemotherapy resistance and poor patient outcomes.

In this review, we summarize and discuss the emerging evidence linking intracellular epigenetic remodeling to the defining features of persister cells in HGSOC. We also address the novel therapeutic strategies aimed at targeting the epigenome of HGSOC to eradicate persister cells and prevent disease recurrence.

### The concept of persistence in MRD

The term persistence is used in both bacteriology and oncology to denote survival without permanent resistance [27]. Persister cells in cancer can be defined by the presence of underlying non-mutational biological mechanisms that mediate drug tolerance, acting as a transition state that is associated with cell survival. Persister cells, synonymously referred to as DTP cells, remain viable in the presence of treatment and may resensitize once the therapeutic intervention is withdrawn. Persister-derived populations can later acquire additional alterations that consolidate stable resistance and lead to refractory disease [28].

One way to understand persister states is through the analysis of tumors after the major insults derived from drug exposure as a representation of MRD. Commonly, tumors are assessed pre- and post-neoadjuvant chemotherapy treatment (NACT) for genomic and non-genomic changes, further revealing drivers or associations with treatment resistance and MRD [24, 29–31]. In HGSOC, recent multiomic approaches have started to elucidate the likelihood of both sub-clonal predisposition to chemotherapy resistance driven by genetic events, or refractory phenotypes [25, 32], and non-genetic mechanisms revealing heterogeneous states of persistence (Fig. 1) [33]. Another concept associated with therapeutic resistance includes the presence of cancer stem cells (CSCs), quiescent or dormant cells. The first evidence in ovarian cancer that a subgroup of neoplastic cells within a tumor had an increased ability to self-renew and sustain tumorigenesis was in 2008 [34]. The concept of persister cells is generally broader but may also include CSCs that possess epigenetic plasticity driven by enhancer rewiring and activation of developmental transcription factor (TF) programs [31, 35, 36]. CSCs are specifically defined by their capacity for self-renewal, but lineage tracing has identified both cycling and non-cycling lineages of persister cells with distinct transcriptional and metabolic features [26, 37]. Several markers of cancer stemness have also been identified in DTP populations, including aldehyde dehydrogenases [38], which detoxify reactive oxygen species, and the CSC surface markers CD133 and CD24 [39]. DTPs have also been reported to share features with therapy-induced senescent populations [40, 41] but diverge in key mechanisms. While senescent cells enter an irreversible, primarily G1/S phase, cell cycle arrest and possess a pro-inflammatory senescence-associated secretory phenotype (SASP), DTPs have been shown to repress interferon/SASP programs and retain the ability to proliferate after drug withdrawal [42]. Diverse drug-resistant mechanisms are believed to be employed by DTP-derived cells, supporting the importance of targeting DTP cells before they acquire irreversible resistance to enhance the durability of responses to cancer treatments.

**Figure 1.**
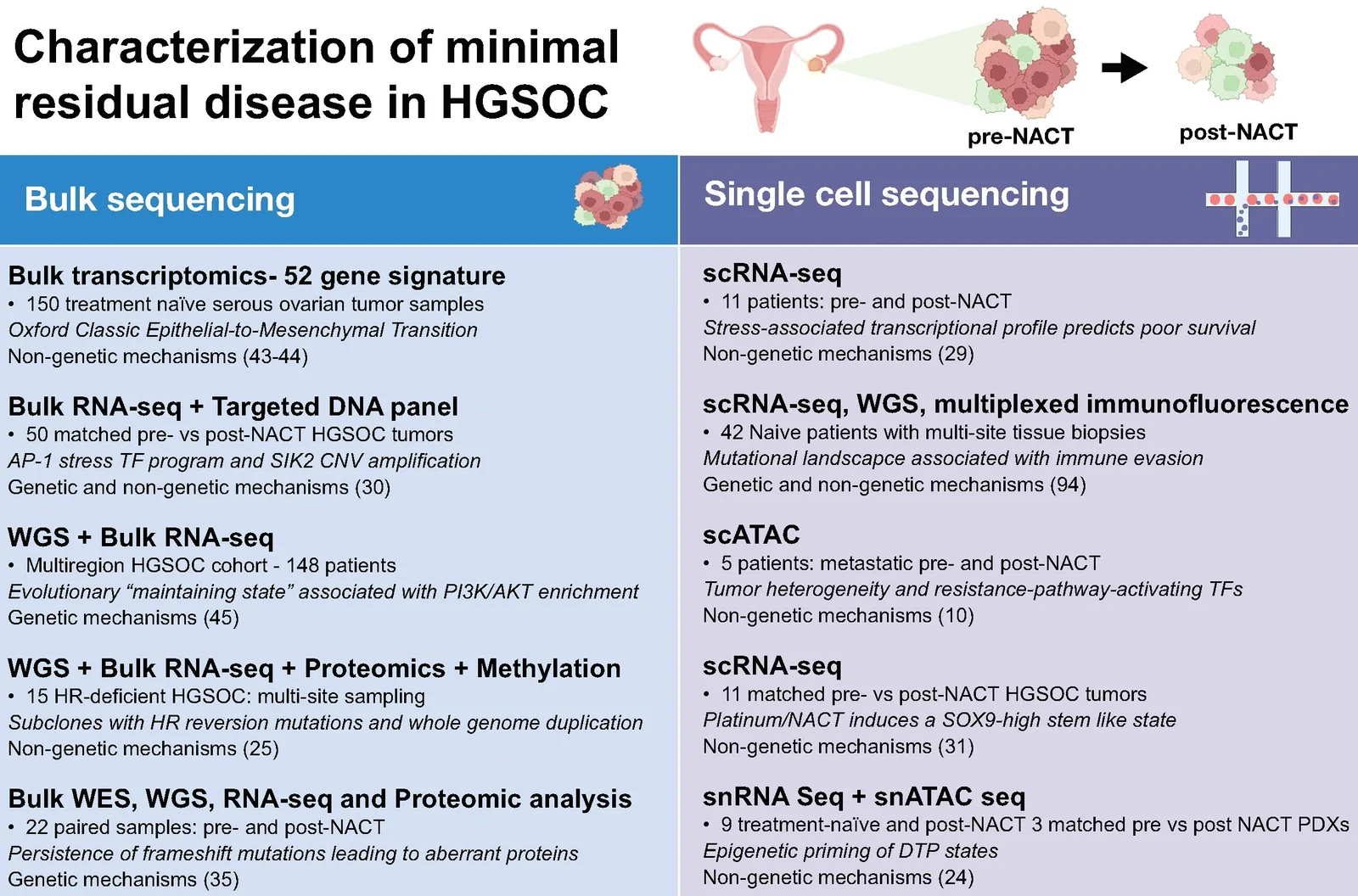
Characterization of MRD in HGSOC using bulk and single-cell sequencing approaches via pre- and post-NACT.

### Emergence of DTPs in HGSOC

In the context of ovarian cancer, bulk transcriptional profiling approaches have already revealed cellular states associated with poor prognosis, such as epithelial-to-mesenchymal (EMT) signatures [29, 43, 44], indicating that cellular heterogeneity could play an important role in defining predisposition for resistance. In this regard, single-cell analyses have helped distinguish between sensitive and therapy-tolerant cells that can coexist in bulk tumor populations and revealed three non-mutually exclusive models of persistence: A) pre-existing persister states; B) persister adaptation; and C) epigenetic priming of persistence (Fig. 2).

**Figure 2.**
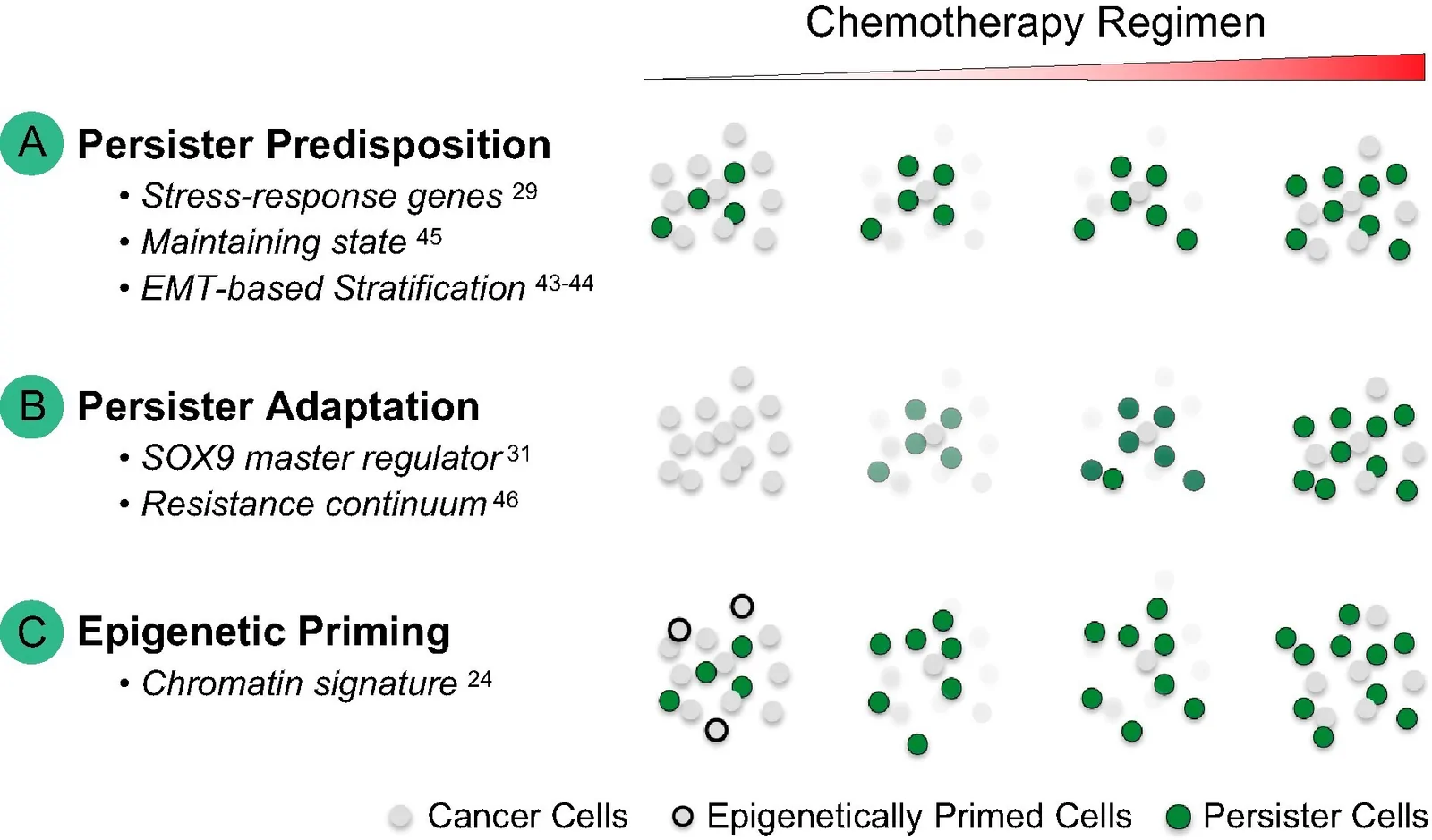
Models of persistence in ovarian cancer.

According to the pre-existing persister-state model, a subset of therapy-tolerant malignant cells exists before treatment onset and is subsequently selected by chemotherapy (Fig. 2A). Genomic evolution and longitudinal transcriptomic profiling support this theory of ovarian cancer persistence. Zhang *et al*. identify a stress-associated malignant cell state that exists in a subset of cancer cells before chemotherapy and becomes sub-clonally enriched during platinum-taxane chemotherapy treatment, consistent with selection of pre-adapted cell states [29]. Similarly, in Lahtinen *et al*., patient tumors were stratified by the presence of stable resistant clones that were found to be pre-existing and selected by platinum-taxane chemotherapy. Notably, this evolutionary “maintaining” state was enriched in PI3K/AKT signaling and detectable after chemotherapy and at relapse, supporting that genomic alterations persist across treatment exposure [29, 45].

Additional evidence points to a resistance continuum model in which cells that have altered chromatin are selected to persist, but treatment further induces changes in chromatin that enable DTP adaptation (Fig. 2B) [29, 46, 47]. This model was demonstrated in ovarian cancer, by which pre-existing variability in epigenetic and transcriptional states enables subpopulations of treatment-naïve malignant cells to tolerate treatment with the PARPi olaparib. Progressively higher doses in the tolerant populations *in vitro* result in increasing fitness along a resistance continuum as the DTPs epigenetically fine-tune stress-adaptative mechanisms [29, 46]. Consistent with this adaptation model, recent longitudinal single-cell studies of matched pre- and post-NACT HGSOC tumors have reported that SOX9 becomes induced in cancer cells following platinum-based chemotherapy, marking a malignant epithelial substate with stem-like and stress-adaptive transcriptional features [29, 31]. This is consistent with the already established role of SOX9 as a TF mediating cell-state determination and EMT regulation embryonically and in adult tissues [48]. Together, this theory of persistence suggests that treatment can actively push surviving cells into a tolerant state rather than merely selecting for fixed resistant clones.

Recent approaches profiling the epigenome and transcriptome of HGSOC patient tumors at a single cell resolution helped determine if features of persister cells comprising residual disease could be identified in treatment-naïve tumors (Fig. 2C) [24]. In this cohort, patients who developed carboplatin/paclitaxel chemotherapy resistance had tumors comprised of cells that differed in their chromatin accessibility compared to patients who were sensitive to chemotherapy. Even before chemotherapy exposure, these cells possess an intrinsic epigenetic program consisting of open chromatin regions where TFs and other chromatin remodelers can bind to drive the expression of the adaptive signaling pathways promoting cell survival. These findings align with the model of epigenetic priming, in which the dysregulation of open chromatin features of DTPs promotes a cell survival fate [24, 49]. These cells are then “poised” by a specific epigenetic program that renders them more susceptible to stable resistant alterations that can persist and be propagated [24, 39].

Taken together, current evidence suggests that DTP emergence in HGSOC may not be explained by a single mechanism but instead reflects the interplay of pre-existing epigenetically primed survival programs, therapy-driven adaptive remodeling along a continuum of tolerant states, and stable genomic or transcriptomic features that can be detected after treatment and at relapse.

### Epigenomic drivers of persister phenotypes

Persistence represents a highly plastic state enabling cancer cells to tolerate cytotoxic stress, establish MRD, and seed subsequent outgrowth of resistant cells. To mitigate the killing efficacy of targeted therapies, these DTP cells have various adaptations, including changes to their cell cycles, metabolism, stress-adaptive responses, and communication with the tumor microenvironment.

#### Quiescence

Across cancers, one mechanism of adaptation of DTPs that enables their survival in the face of therapies that target rapidly dividing cells is their ability to exit the cell cycle. Distinct from genetic resistance, quiescence is reversible as DTPs gradually regain their ability to proliferate after drug withdrawal. This state can in part be explained by promoter hypermethylation and enrichment of repressive histone marks (H3K27me3, H3K9me3), and loss of activating marks (H3K4me3, histone acetylation) at cell-cycle and DNA-repair genes [24, 50–52]. Histone demethylases such as KDM5A/B and deacetylases such as HDAC1/2 are the key enforcers of this landscape, and their inhibition has been shown to reduce the fraction of cells in the DTP state [24, 39]. In advanced treatment-refractory breast cancers, the ability to generate DTPs through docetaxel is linked to the activity of the G1/S cell-cycle regulator p27 (encoded by *CDKN1B*), which inhibits the cyclin D-CDK4/6 and cyclin E-CDK2 complexes [50, 53]. CDKN1B protein stabilization is mediated by upstream AKT1 phosphorylation, resulting in an almost five-fold increase of protein but consistent mRNA levels in DTPs [50]. Evidence also shows that CDKN1B suppression reduces the accumulation of induced DTPs. In attempts to target this mechanism, AKT inhibitor MK2206, treatment two days following docetaxel treatment limits the growth of DTPs [50, 54]. This and other studies have explored the relevance and targetability of the PI3K/AKT/mTOR pathway with promising results even beyond DTPs [50, 53, 54]. Similarly, direct DNA modifications by the methylcytosine dioxygenase TET2, a member of the Ten-eleven translocation (TET) family, have been identified as a key regulator of chemo-resistant slow-cycling cancer cells. In multiple cancer types, by oxidizing 5-methylcytosine (5mC) to 5-hydroxymethylcytosine (5hmC), TET2 represses the expression of E2F-related cell proliferation pathways [55]. In addition to entry into the slow-cycling or dormant state, DTPs also have the ability to reversibly regain their proliferative capacity. One mechanism by which this may occur in DTPs is the inhibition of JAK/STAT signaling, which is linked to expansion of stem cell populations during development [56–58].

In the context of HGSOC, the transcription factor NFATC4 (NFAT3) is activated by chemotherapy treatment and demonstrated to induce a non-dividing state that confers resistance to platinum and taxane regimens. Downstream of NFATC4 is paracrine signaling by follistatin (FST), which is secreted into the tumor microenvironment to induce the quiescent state in neighboring cancer cells [59, 60]. In nonmalignant context, SOX2, SOX9, and ZEB1 have been shown to be key in embryonic development and cell-fate decisions, whereas the E2F family are transcriptional regulators of cell cycle [31, 48, 61–66]. Chromatin accessibility profiling of HGSOC has also defined DTPs by their enrichment of several quiescence-associated TFs and chromatin remodelers, including SOX2 [63, 67], SOX9 [31, 64, 68], E2F1/7 [61], and ZEB1 [56]. A subpopulation of SOX9-expressing cells is upregulated by chemotherapy treatment in HGSOC patient tumors and cell lines and is also detectable in primary tumors. SOX9 promotes cancer stemness features, including transcriptional plasticity and platinum resistance *in vitro* and *in vivo* [31]. Further supporting low proliferative capacity in MRD DTPs, Croft *et al*. identified the closure of binding sites for E2F2 and E2F4 transcriptional drivers in post-treated HGSOC [10]. In parallel, work on PARP inhibitor-treated HGSOC shows that sublethal olaparib induces polyploid giant cancer cells (PGCCs) with senescence-like features [69]. Following drug withdrawal, PGCCs escape cell cycle arrest via atypical divisions to generate mitotically competent, PARP inhibitor-resistant progeny. Zhang *et al*. show that the antiprogestin contraceptive drug, mifepristone, can block the formation and survival of PGCCs by promoting apoptosis of cells undergoing aberrant endoreplication [69]. In both organoid and patient-derived xenograft (PDX) models of acquired olaparib resistance, mifepristone enhanced the antitumor activity of olaparib. Further in PARPi-naïve models, mifepristone alone induced modest tumor growth inhibition, whereas the combination of mifepristone and olaparib produced substantially greater tumor suppression than either agent alone. These findings identify polyploid persistence driving PGCC formation as another non-genetic route to survival in ovarian cancer that can be targeted to prevent disease recurrence.

#### Metabolism and stress adaptations

Adaptation to stress from cytotoxic treatments and hypoxic, nutrient-deprived conditions represents another mechanism of cancer cell persistence [26]. To cope with these stressors, DTPs can upregulate their integrated stress response and antioxidant systems such as ATF4-regulated transcriptional programs that promote autophagy, nutrient scavenging, and redox homeostasis [70]. Persister cells can shift their metabolism toward oxidative phosphorylation and utilize alternative nutrients in response to environmental metabolite levels. These metabolic strategies, including fatty acid β-oxidation [71, 72] and autophagy [73], generate reactive oxygen species and initiate the activity of antioxidant defense signaling pathways regulated by master sensors of redox homeostasis, including Nuclear Respiratory Factor 1 and 2 (NRF1 and NRF2). NRF2 is activated in dormant disease, including in HGSOC, and linked to metabolic reprogramming and cancer recurrence [74]. Working with its transcriptional binding partner CBP/p300, NRF2 remodels enhancer landscapes around stress-responsive regions, establishing a metabolic-epigenetic loop [75, 76].

In metastatic HGSOC tissues, post-chemotherapy states are characterized by open chromatin regions enriched for resistance and stress-associated regulators’ binding motifs [10]. At the single cell level, these stress-adaptive responses are associated with poor patient outcomes and are directly regulated by TFs and chromatin remodelers that define DTPs in HGSOC [24]. França *et al*. show that adaptation to increasing doses of PARP inhibitors *in vitro* is accompanied by progressive enrichment of oxidative phosphorylation, lipid metabolism, and stress regulators (AP-1, NRF2, and ATF4) [46]. They also suggest that the adaptation to chemotherapy develops through trajectories of cell-state transitions accompanied by a progressive increase in cell fitness as more of a continuum. These chemo-resistant adapted states acquire new metabolic dependencies that can be exploited for combination therapy, such as the inhibition of glutathione metabolism [77].

#### Epithelial-to-mesenchymal plasticity

Persister populations across cancers show partial EMT signatures by which epithelial cells adopt mesenchymal features such as increased motility and invasion [78, 79]. In HGSOC, transcriptomic analyses across multiple independent patient cohorts demonstrate that EMT-high tumors exhibit significantly worse overall survival than EMT-low tumors, highlighting EMT as both a prognostic biomarker and potential therapeutic target [43, 44]. Molecular EMT-based risk stratification identifies patients with poor clinical outcomes and suggests that these tumors may benefit from therapeutic strategies targeting EMT programs [43, 44]. In these cells, the expression of the demethylase KDM6A is reduced, such that adhesion genes are silenced via promoter H3K27me3 to initiate a pre-metastatic cascade [80]. EMT-associated TFs such as ZEB1, TWIST1, and SNAI1 promote the expression of mesenchymal markers like vimentin and TGF-β/Notch target genes [62, 81]. ZEB1-enriched mesenchymal cancer cells also acquire a dependency on the lipid peroxide detoxifier GPX4, which prevents ferroptosis by reducing byproducts of lipid peroxidation [78]. Hence, therapies targeting the ZEB1-GPX4 axis result in selective persister cell ferroptotic death, including in HGSOC, representing a therapeutic strategy to prevent cancer relapse [82, 83].

Böpple *et al*. further link an EMT state in cisplatin-tolerant HGSOC cells to the AP-1 family TF ATF3. *In vitro*, ATF3-high DTP subpopulations show enhanced invasion and chemotolerance. Moreover, knockdown of ATF3 restores cisplatin sensitivity [84]. Single-cell data from patient tumors reveal *ATF3*-expressing clusters even prior to treatment and predict poor outcomes, supporting an ATF3-mediated mechanism of EMT in DTPs.

#### Altered communication with the tumor microenvironment

Lastly, DTPs also exhibit altered communication with the TME, beyond the known mutational alterations that show an important impact in shaping immune response. Chemotherapy treatment in HGSOC initially induces an enrichment of tumor and stromal cell populations expressing interferon and inflammatory signaling associated with long-interspersed repeat element 1 (LINE-1) retrotransposon expression and innate immune activation [85]. However, across cancers, persisters eventually appear to downregulate inflammatory and interferon signaling while maintaining or enhancing interactions with the TME that support survival [42, 86]. It has been shown that immunosuppression by DTPs is mediated by increased repressive histone marks (H3K9me3) enriched in LINE-1 loci. Disrupting this repression with DNA demethylating agents and HDAC inhibitors unleashes LINE-1 transcription to activate antiviral and interferon responses and selectively kill persisters [52]. Others show that persister cells can also stably repress interferon and SASP pathways via H4K20me3, distinguishing them from senescent subpopulations [42].

In HGSOC, the surrounding omentum and ascites provide rich stromal and immune niches that are permissive for persistence of MRD. Malignant ascites is an abnormal accumulation of fluid in the peritoneal cavity that commonly occurs in advanced cancers, including HGSOC, and facilitates the spread of tumor cells as spheroids throughout the abdomen, promoting peritoneal dissemination and metastatic progression [87]. This process occurs within a unique intraperitoneal environment, where poorly perfused and irregularly vascularized tumor regions give rise to hypoxic and nutritionally deprived conditions, including reduced glucose availability. Together, these metabolic stressors, combined with continuous shedding of tumor cells into the peritoneal cavity, promote the formation of multicellular spheroids that serve as the primary vehicle for peritoneal dissemination and metastatic seeding [88]. In addition to metabolic stress, tumor cells within ascites are exposed to distinct mechanical pressures and forces, including fluid shear stress, which can influence spheroid formation, cytoskeletal remodeling, survival, and metastatic potential [89]. Although ascites remains relatively understudied, it represents a dynamic and heterogenous microenvironment that may contribute to the development and/or maintenance of DTPs. Functionally, patient ascites-derived epithelial (EPCAM+) spheroids, particularly those enriched in chemo-resistant patients, demonstrated tumor-initiating capacity *in vivo* and recapitulated peritoneal dissemination and ascites reformation, supporting the concept that ascites may function as a reservoir of tumor-propagating cells under therapeutic pressure [87]. In contrast, mesenchymal-like adherent populations displayed reduced tumorigenicity and increased drug sensitivity, suggesting that therapeutic pressure may select for epithelial, stem-like ascites populations with enhanced survival capacity [87]. Together, these findings support a model in which malignant ascites is not merely a passive reservoir of exfoliated tumor cells, but rather a biologically active niche that both selects for and potentially promotes drug-tolerant cellular states through mechanical and environmental cues. In this context, ascites-derived epithelial spheroids may represent a permissive environment in which environmental stressors contribute to the emergence of relapse-initiating, therapy-adapted tumor populations in ovarian cancer, including DTP-like states.

As for immune and microenvironment context, cytokines, including IL-6 and TGF-β, secreted by cancer-associated fibroblasts activate TFs, including STAT3, SMAD, and YAP/TAZ, in tumor cells, reinforcing quiescence, EMT, and stress programs [90]. Beyond stromal-derived cytokines, HGSOC cells also actively shape the immune microenvironment through tumor-derived IL-4. Leveraging a spatial functional genomics screen, Mollaoglu *et al*. show that tumor-derived IL4 directs the formation of localized immunosuppressive macrophage-rich niches that promote resistance to anti-PD1 therapy [91]. Epigenetic silencing of proinflammatory chemokines such as CXCL9 and CCL5 in tumor cells via the histone methyltransferase enhancer of zeste homolog 2 (EZH2)-mediated H3K27me3 and DNMT1-mediated DNA methylation further contributes to a “cold” microenvironment with limited T-cell infiltration [92, 93]. Recent spatial multi-omics studies further demonstrate that immune suppression is highly heterogeneous across HGSOC, varying by both metastatic site and underlying mutational processes, with distinct mechanisms of immune evasion emerging in different tumor niches and genomic subtypes [94].

### Epigenetic therapies targeting persister cells

Considering the role of persister cells in chemoresistance and cancer recurrence, there is a strong rationale for the development of therapies that target epigenetic dependencies associated with residual, stress-adapted tumor states. Importantly, current clinical testing of epigenetic therapies in HGSOC has focused exclusively on the recurrent or platinum-resistant setting, in which these agents are used to treat visible recurrent disease rather than MRD after initial therapy. Epigenetic combinations will need to show efficacy in treating recurrent disease before they can be investigated in frontline combinations or as maintenance therapy.

In combination with platinum and taxol-based chemotherapy regimens that most tumor cells are sensitive to, targeting the epigenetic hallmarks of DTPs presented in this review represents a strategy to prevent cancer relapse (Table 1). Epigenetic therapies aimed at inhibiting DNA methyltransferases and chromatin remodelers have undergone significant preclinical and clinical evaluation across multiple cancer types. However, since chromatin dynamics are critical for physiologic and developmental processes throughout the genome, the challenge with their implementation is related to their specificity to target cancer cells exclusively. To date, few clinical trials for epigenetic drugs have been investigated in HGSOC.

**Table 1.** Clinical trials of epigenetic agents targeting DTP features in HGSOC

Class	Treatment	Primary target	Functional effects	Evidence for HGSOC	References
DNMT inhibitors	Decitabine, guadecitabine	DNMT1, DNMT3A/B	Repression of apoptotic and immune genes	Tested with carboplatin and PARP inhibitors in platinum-resistant HGSOC	[95, 96]
HDAC inhibitors	Entinostat, vorinostat	Class I/II HDACs	Genetic instability and repression of immune genes	Sensitizes HR-proficient HGSOC to PARP inhibitors	[97]; NCT03924245
BET bromodomain inhibitors	ZEN-3694, BMS-986 158, I-BET-762, JQ1	BRD2/3/4	Super-enhancer driven stress and EMT programs	HGSOC shows a dependency to SE, BET inhibitors sensitize to platinum/PARP inhibitors preclinically	[98, 99, 100]; NCT05950464 NCT04840589
CBP/p300 HAT inhibitors and dual BET inhibitors	NEO-2734, inobrodib, A-485	CBP/p300	Stress-adaptative enhancers	NRF2–CBP/p300 activity at enhancers for stress associated genes in HGSOC	NCT05488548
EZH2/EZH1 inhibitors	Tulmimetostat, tazemetostat	PRC2/EZH2	EMT plasticity	Synthetic lethality in advanced ARID1A-mutated ovarian clear cell carcinoma	NCT04104776

In ovarian cancer patients, these agents are currently being investigated for treatment of recurrent disease in the platinum-resistant setting.

BET proteins (BRD2/3/4) and CBP/p300 cooperate at super-enhancers to maintain antioxidant, EMT, and metabolic programs in cycling persister cells. BET inhibition has been shown to selectively eliminate DTPs that have re-entered the cell cycle by collapsing redox-regulating transcriptional networks [98]. Further, the CREBBP/EP300 inhibitor inobrodib and BRD2/4 inhibitor I-BET-762 have been identified as pharmacological options to overcome HGSOC carboplatin resistance *in vitro* [24]. These findings align with previous studies showing that BET inhibitors synergize with carboplatin [100, 101] and PARP inhibitors in chemoresistant states [99, 102] and also suggest that the histone acetyltransferases CREBBP/EP300 represent promising therapeutic targets in ovarian tumors, as has been described in multiple cancers [103] and ovarian cancer [24].

Other DTP vulnerabilities include DNMTs, EZH2, and the H4K20 methyltransferases KMT5B/C, which repress apoptotic and interferon pathways [42, 52]. Recently, combined DNMT and PARP inhibition in ovarian cancer was shown to induce a STING-dependent “pathogen mimicry response,” mediated through the nucleic-acid sensor ZNFX1, which activates interferon and inflammasome signaling [104]. In this context, ZNFX1 serves as a tumor suppressor, and its expression was significantly correlated with an increase in overall survival of patients with treatment-resistant ovarian cancer. This reinforces the importance of epigenetic-priming strategies that aim to re-activate antiviral/innate immune programs in residual disease and the need for identification of candidate biomarkers linked to patient responses such as ZNFX1.

Notably, a signature of DTPs or MRD could identify the subset of patients most likely to benefit from epigenetic therapy. This could enable future studies that test frontline epigenetic chemotherapy combinations to reduce the emergence of MRD, or maintenance epigenetic therapy after initial treatment responses to delay recurrence.

### The epigenome as a clinical biomarker for persistence

Given the high recurrence rates observed in HGSOC, early identification of patients harboring tumors with cells that give rise to persisters could facilitate personalized treatment strategies aimed at pre-emptively targeting residual disease. Similar approaches have already been developed to identify refractory clones by combining whole genome sequencing and targeted sequencing in cell-free DNA [105, 106]. Similar to refractory clones, DTPs are highly heterogenous and adaptable, making identification of consistent druggable targets across patients immensely difficult [28, 105]. This section outlines how emerging research platforms can be leveraged to discover and validate novel biomarkers of DTPs that may ultimately inform clinical decision-making.

Cells in the DTP state may be identified by their chromatin accessibility features using single-cell transcriptomic and epigenomic profiling (scRNA seq + scATAC seq) [24]. Emerging transcriptome-recording approaches such as TimeVault may complement these single-cell approaches by enabling retrospective reconstruction of transient pre-treatment and early-treatment transcriptional states that give rise to persister phenotypes [107]. Additional single-cell chromatin assays such as scCUT&TAG can help to resolve the histone marks (e.g. H4K20me3 over SASP genes) at loci that distinguish persisters from sensitive populations [108].

Spatial transcriptomic and epigenomic approaches will help to further define DTP states at a high resolution by mapping persister cell signatures within their tissue contexts. Characterizing the spatial relationships between the tumor-stroma interfaces will identify if there are specific niches that cultivate the development of DTPs and help to identify which interactions between DTPs and other cell types are the key drivers of resistance phenotypes [109–111]. The emergence of spatial epigenomic profiling via spatial ATAC and spatial CUT&TAG [112, 113] will improve the understanding of the dynamic changes in DTP chromatin throughout the course of therapeutic regimens and enable the tracking of clonal lineages within heterogenous cancer cell populations. Although these single-cell and spatial platforms are highly informative for discovery, their current cost, turnaround times, and technical complexity limit their inclusion in current clinical workflows. Their primary value at present lies in advancing the classification and understanding of DTP heterogeneity, with the goal of identifying robust biomarker signatures that can be translated to stratify patient care.

Amongst the available technologies, methylome profiling of patient tumors represents the most clinically accessible approach for epigenetic biomarker development [114]. Based on methylation profiles and predicted TF activities, this data could be used to infer which chromatin regions are likely accessible or repressed and which TFs are binding to the accessible sites to drive mechanisms of persistence. Where deeper characterization is needed, long-read epigenomic profiling of 5mC and 5hmC could allow for improved resolution of epigenetic alterations within regulatory and repetitive genomic regions that are difficult to interrogate using conventional short-read approaches [115]. Other long-read sequencing approaches such as FIBER-seq may enable the development of single nucleotide maps comprised of nucleosome positioning and chromatin architecture information that can distinguish persister cell populations [116].

Collectively, the integration of these complementary platforms, including high-resolution single-cell and spatial discovery tools, to more clinically scalable methylome and long-read approaches will elucidate the nature of persistence and define druggable targets to develop more unified patient treatments.

### Concluding remarks and perspective

DTP populations are thought to serve as a reservoir of cells from which genetically resistant lineages emerge in HGSOC. Advances in single-cell epigenomic profiling and spatial characterization of heterogeneous patient tumors now enable more precise identification of these persister cells and their epigenetic features. We propose that combining current standard of care treatments with epigenetic agents targeting DTP subpopulations will deplete this reservoir and limit the emergence of resistant clones. Exploiting the epigenetic vulnerabilities that sustain persistence represents a rational and actionable strategy to delay or prevent disease recurrence.

## Data Availability

No new data were generated or analysed in support of this research.
